# Selection against Aerial Dispersal in Ants: Two Non-Flying Queen Phenotypes in *Pogonomyrmex laticeps*

**DOI:** 10.1371/journal.pone.0047727

**Published:** 2012-10-23

**Authors:** Christian Peeters, Roberto A. Keller, Robert A. Johnson

**Affiliations:** 1 Laboratoire Ecologie & Evolution, Centre National de la Recherche Scientifique unité 7625, Université Pierre et Marie Curie, Paris, France; 2 Instituto Gulbenkian de Ciência, Rua da Quinta Grande, Oeiras, Portugal; 3 School of Life Sciences, Arizona State University, Tempe, Arizona, United States of America; University of Arizona, United States of America

## Abstract

The South American seed-harvester ant *Pogonomyrmex laticeps* has dimorphic queens: ergatoid (permanently wingless) and brachypterous (short, non-functional wings). Surveys in western Argentina indicated that colonies near Chilecito, La Rioja Province, produced only ergatoid queens, while those near Punta Balasto, Catamarca Province (263 km away), produced only brachypterous queens. Brachypterous queens were significantly larger than ergatoid queens for 10 of 11 external characters, but both phenotypes had comparable reproductive potential, i.e., a spermatheca and a similar number of ovarioles. Using normal winged queens of the closely related *P. uruguayensis* for comparison, we determined that both queen phenotypes in *P. laticeps* had a full set of dorsal thoracic sclerites, albeit each sclerite was much reduced, whereas workers had a thorax without distinct dorsal sclerites. Sclerites were fused and immobile in ergatoid queens, while they were separable and fully articulated in brachypterous queens. Both phenotypes lacked the big indirect flight muscles, but brachypterous queens retained the tiny direct flight muscles. Overall, this dimorphism across populations indicates that there are alternative solutions to selective pressures against flying queens. We lack field data about colony founding strategy (independent or dependent) for either queen phenotype, but colonies at both sites produced numerous gynes, and we infer that all foundresses initiate colonies independently and are obligate foragers.

## Introduction

The sporadic but recurrent loss of flight in insects is a tantalizing evolutionary problem. Ability to fly is a tremendous adaptation that enhances dispersal, location of scattered resources (food and mates), and avoidance of predators. In species such as bees and dragonflies, flight is an integral part of daily life, while in other insects it only serves as a mechanism for dispersal. In the latter case, flight can be lost or it can occur as a polymorphism, with many examples in the orders Orthoptera, Hemiptera, Hymenoptera, and Coleoptera [1]. Solitary insects with a flight polymorphism often display a trade-off between dispersal capability and fecundity, with the flightless phenotype having a higher fecundity than the flight-capable phenotype [2], [3], [4].

Ants are an example of insects in which flight only functions for dispersal. Young queens and males fly from their natal nests to mate; queens then locate a new nesting site and shed their wings. Unlike other eusocial Hymenoptera, ants also have a permanently wingless worker caste, such that both winged and wingless females are produced in the same colony. Nevertheless, even this highly reduced function of flight has been lost numerous times and ergatoid (permanently wingless) queens occur in species belonging to most subfamilies of ants [5]. It is generally thought that flight-capable queens are selected against when the energetic or mortality costs of long-distance dispersal outweigh the benefits of colonizing disjunct habitats.

The seed-harvester ant genus *Pogonomyrmex* (subfamily Myrmicinae) exhibits a diversity of queen phenotypes that parallels that found within all ants. Nine species produce ergatoid queens, three species produce both winged and ergatoid queens, and three species produce brachypterous queens (having rudimentary or abnormally short, non-functional wings) [6,7,8,9,10,11, R.A. Johnson, unpublished data]. Additionally, Kusnezov [10] reported the occurrence of two types of flightless queens (ergatoid and brachypterous) in *P. laticeps* Santschi, 1922, but he did not provide additional information on these queens. *Cataglyphis floricola* is the only other ant species known to have both ergatoid and brachypterous queens [12].

This study expands on the observations of Kusnezov [10] by documenting morphology, wing musculature, reproductive potential, gyne production, and geographic variation in occurrence of the two queen phenotypes in *P. laticeps*. We use indirect evidence to speculate on the colony founding strategy used by *P. laticeps*.

**Figure 1:**
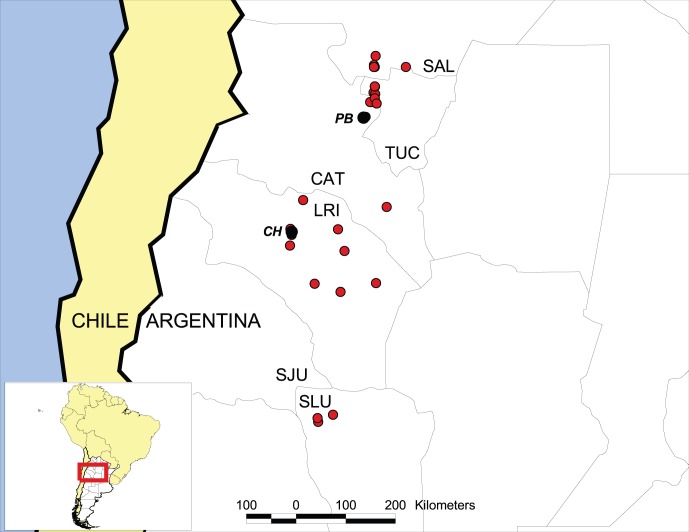
Distribution map for *Pogonomyrmex laticeps*. The two study sites are shown by black symbols, *CH* = Chilecito, *PB* = Punta Balasto; red symbols = localities where *P. laticeps* has been collected. Provinces in Argentina are as follows: SAL = Salta; TUC = Tucumán; CAT = Catamarca; LRI = La Rioja; SJU = San Juan; SLU = San Luis.

## Methods

### Study Sites

We studied *P. laticeps* during February 2010 at two sites in western Argentina: (1) Ruta 40 at 3.8 km north of Punta Balasto, Catamarca Province (26°59′S, 66°10′W; elevation 2135 m), and (2) Ruta 40 at 11–14 km north of Chilecito, La Rioja Province (29°03′S, 67°28′W; 1035 m). The two sites are separated by approximately 263 km (Figure 1). At both sites, most colonies were collected in disturbed roadside habitats; surrounding areas consisted of Monte Desert woodland. Common perennial plant species at the Punta Balasto site included *Acacia* sp., *Larrea* sp., *Lycium* sp., *Parkinsonia* sp. and *Prosopis* sp., while *Acacia* sp., *Parkinsonia* sp., *Prosopis* sp. and *Bulnesia* sp. were the most common species at the Chilecito site. Soils consisted of deep sand at the Punta Balasto site and rocky, gravelly clay at the Chilecito site.

**Figure 2:**
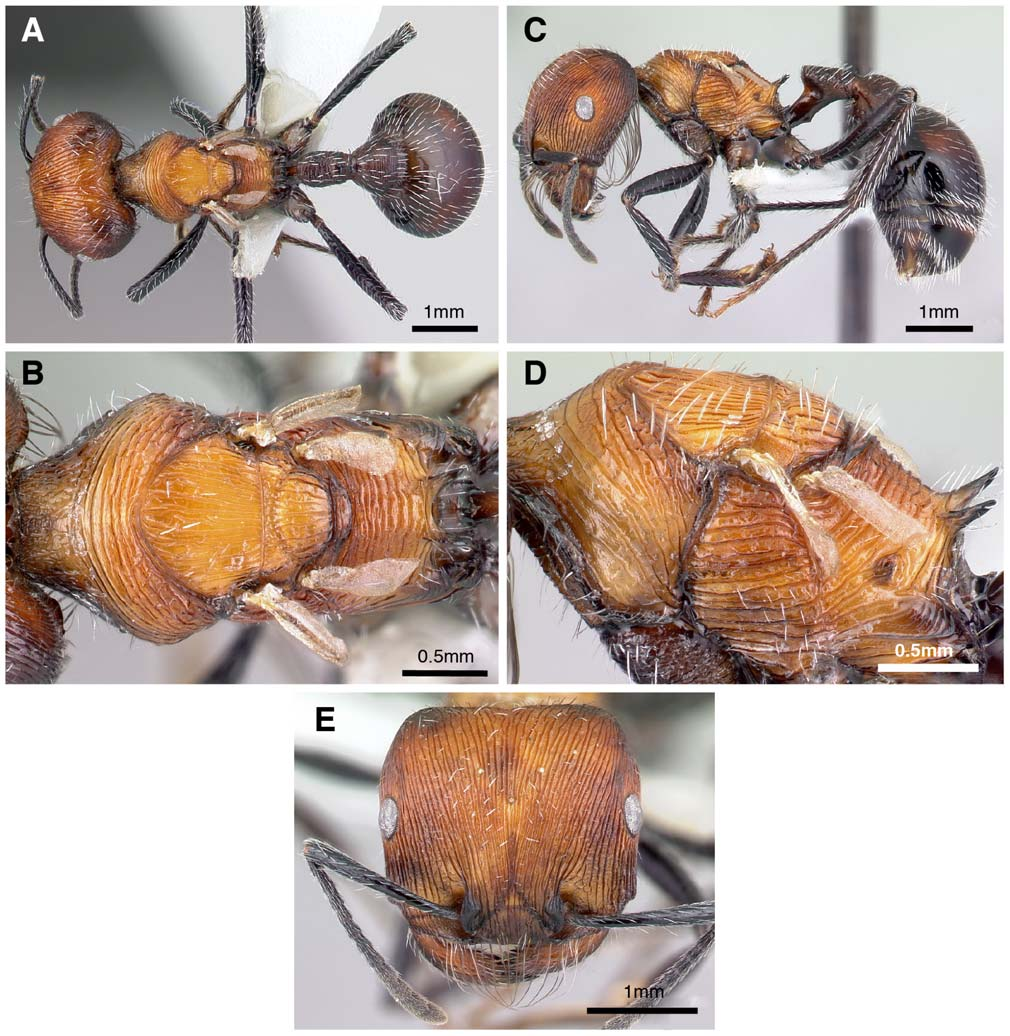
Photographs of *Pogonomyrmex laticeps* brachypterous queen. (A) dorsal view of body, (B) close-up dorsal view of thorax, (C) lateral view of body, (D) close-up lateral view of thorax, (E) frontal view of head. High resolution photos for all castes (Figures 2–4) are also available at http://www.antweb.org/ and http://www.asu.edu/clas/sirgtools/pogonomyrmex/SOUTHAMERICANPOGOS.htm.

**Figure 3:**
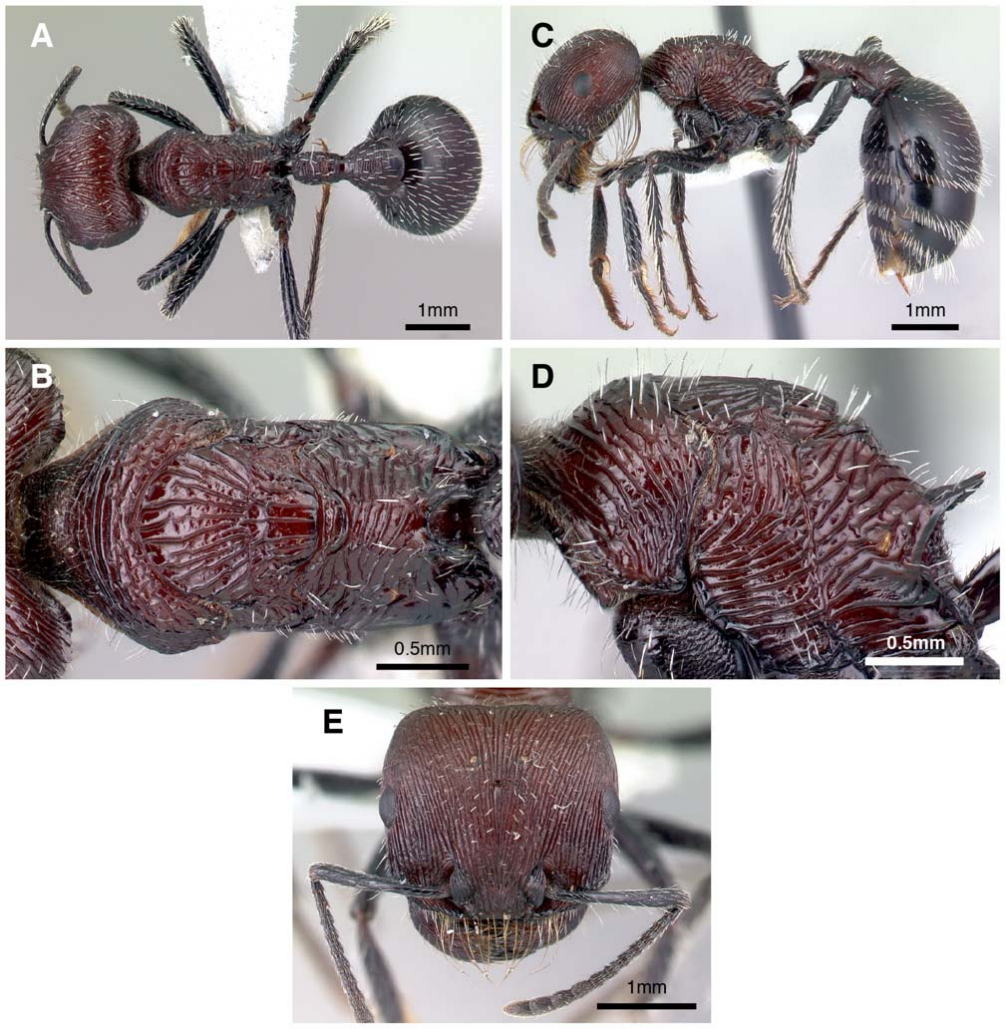
Photographs of *Pogonomyrmex laticeps* ergatoid queen. (A) dorsal view of body, (B) close-up dorsal view of thorax, (C) lateral view of body, (D) close-up lateral view of thorax, (E) frontal view of head.

### Nest Excavations

We excavated 16 nests of *P. laticeps* to determine the number and type of young queens (gynes) that were produced. At the Punta Balasto site, we excavated chambers until no more workers were found, and we revisited nests for 2 subsequent days to dig further around the spots where workers had reopened tunnels. Even after 2–3 days, we do not consider these excavations to be complete. In the Chilecito site, the rocky, gravelly soil made digging difficult. Consequently, we scooped out a 3–4 cm deep depression (15–20 cm in diameter) centered on nest entrances. We then dripped 1–2 liters of water onto each nest, which induced individuals to move upward; surface chambers were excavated approximately 12 h later. Individuals from each nest (including pupae) were examined under a dissecting microscope and identified as worker, ergatoid queen, or brachypterous queen; individuals of each caste were then counted. Myrmicine pupae are not enclosed in a cocoon so gynes could be identified by their tiny wing sheaths and/or ocelli.

**Figure 4:**
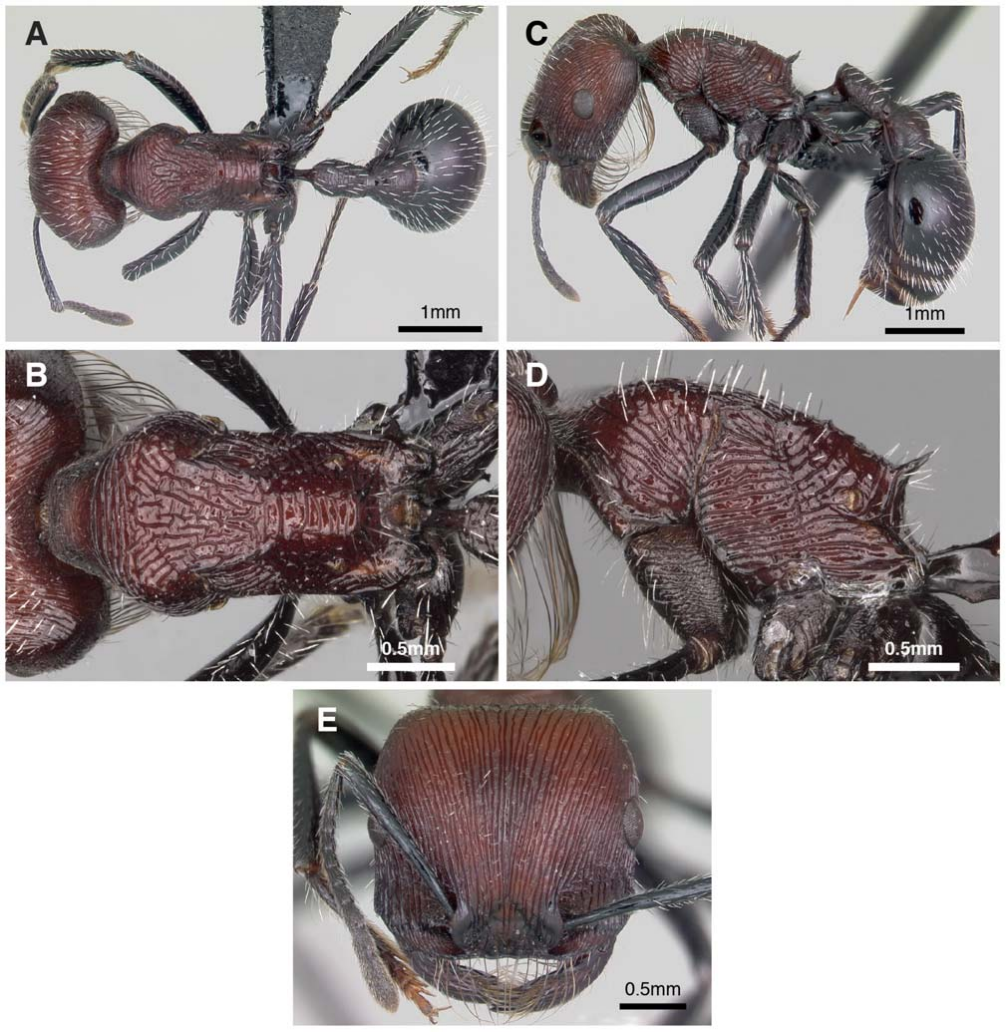
Photographs of *Pogonomyrmex laticeps* worker. (A) dorsal view of body, (B) close-up dorsal view of thorax, (C) lateral view of body, (D) close-up lateral view of thorax, (E) frontal view of head.

**Table 1:** Measurements (in mm; mean ± SE) for the three female castes of *Pogonomyrmex laticeps*.

		Worker +			Ergatoid	Brachypterous	
Character*		Chilecito	Punta Balasto	queen		queen	
**Head width (HW)**		1.85 ± 0.02^d^	2.07 ± 0.03^c^		2.18 ± 0.02^b^		2.39 ± 0.02^a^
**Head length (HL)**		1.74 ± 0.02^d^	1.91 ± 0.03^c^		2.01 ± 0.02^b^		2.14 ± 0.02^a^
**Maximum eye diameter (MOD)**		0.35 + 0.00^c^	0.37 + 0.01^b^		0.39 ± 0.00^b^		0.43 ± 0.00^a^
**Scape length (SL)**		1.34 ± 0.02^c^	1.50 ± 0.02^b^		1.47 ± 0.02^b^		1.56 ± 0.02^a^
Ocellus diameter (OD)		Absent^b^	Absent^b^		0.05 ± 0.00^a^		0.05 ± 0.00^a^
**Pronotal width (PW1)**		1.16 ± 0.01^d^	1.30 ± 0.02^c^		1.39 ± 0.01^b^		1.49 ± 0.01^a^
**Petiole width (PW2)**		0.47 ± 0.01^d^	0.52 ± 0.01^c^		0.56 ± 0.01^b^		0.59 ± 0.00^a^
**Post-petiole width (PW3)**		0.68 ± 0.01^d^	0.78 ± 0.01^c^		0.88 ± 0.01^b^		0.93 ± 0.01^a^
**Thorax length (ML)**	2.13 ± 0.03^d^		2.37 ± 0.03^c^		2.55 ± 0.03^b^		2.69 ± 0.02^a^
**Hind Femur length (HFL)**	1.80 + 0.02^c^		2.09 + 0.03^b^		2.06 + 0.02^b^		2.26 + 0.02^a^
Gastral t**ergite width (TW)**	1.50 ± 0.02^d^		1.74 ± 0.03^c^		2.22 ± 0.02^b^		2.35 ± 0.02^a^
Number of ovarioles	2.00 ± 0.00^b^		2.00 ± 0.00^b^		13.20 ± 0.37^a^		13.00 ± 0.58^a^
Spermatheca	No		No		Yes		Yes

* **HW** – maximum width of the head, positioned in full face view, at a level above the upper eye margin;

**HL** - maximum length of the head, full face view, from the midpoint of the anterior clypeal margin to the midpoint of the occipital margin; **MOD** – maximum diameter of the eye as measured with the head in full lateral aspect; **SL** – maximum length of the scape, including the basal condyle; **OD** – maximum diameter of the anterior ocellus; **PW1–3** – maximum width of pronotum, petiole, and post-petiole, respectively, as seen from above, at right angles to the longitudinal axis of the body; **ML** - diagonal length of the thorax in profile from the anterior pronotal margin to the posterior base of the metapleural lobe; **HFL** – length of the hind femur, measured along the dorsal margin from the articulation with the trochanter to the most distal tip of the femur; **TW** – maximum width of first gastral tergite, as seen from above, perpendicular to the longitudinal axis.

+ **= **For external morphological characters: *n* = 40 workers (15 at Chilecito [CH], 25 at Punta Balasto [PB]), *n* = 25 ergatoid queens (CH), *n* = 30 brachypterous queens (PB); for internal characters: *n* = 8 workers (2 at CH, 6 at PB), *n* = 5 ergatoid queens, *n* = 3 brachypterous queens (see text for details). Significant differences among castes are given by the letters *a-d*: *a* > *b* > *c* > *d*. Groupings are based on univariate F tests within a MANOVA followed by a Duncan’s multiple range test for continuous variables or a Kruskal-Wallis test for discrete variables (see text). **Characters in bold font** were used in the discriminant analysis.

### Morphometric and Anatomical Comparison Across Female Castes

We quantified 14 morphological characters (11 external, three internal) for workers, ergatoid queens, and brachypterous queens. External characters were head width, head length, scape length, maximum eye diameter, diameter of the anterior ocellus, pronotal width, mesosomal length, hind femur length, petiole width, post-petiole width, and width of the first gastral tergite [see also 7]; internal characters were number of ovarioles, presence/absence of a spermatheca, and presence of the flight muscles. The 11 external characters were measured by projecting an image from a dissecting microscope to a video monitor; this image was measured to 0.01 mm using NIH Image (available at http://rsb.info.nih.gov/nih-image/). The three internal characters were examined via dissections under a stereomicroscope. For all analyses, workers from the two sites were treated as separate groups in order to assess geographical variation in worker size.

**Figure 5:**
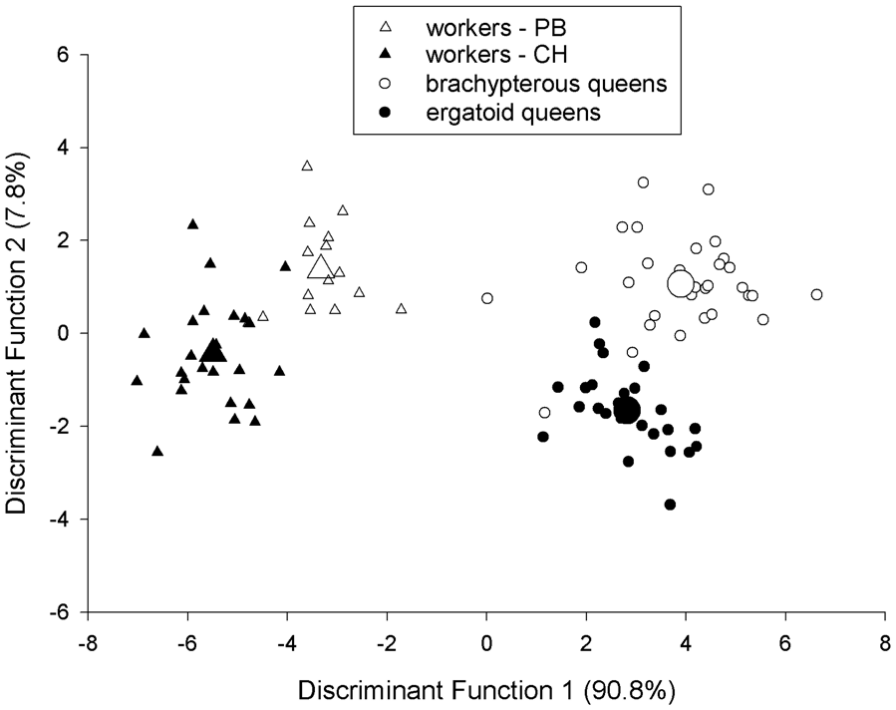
Discriminant scores for the three female castes of *Pogonomyrmex laticeps*. Scores of all individuals (*n* = 40 workers, *n* = 25 ergatoid queens, *n* = 30 brachypterous queens) are projected onto the two-dimensional space defined by discriminant functions 1 and 2. The larger symbols for each caste are the unstandardized canonical discriminant functions evaluated at the group means (centroids). PB = Punta Balasto, CH = Chilecito.

**Table 2:** Standardized coefficients for canonical discriminant functions for workers, ergatoid queens, and brachypterous queens of *Pogonomyrmex laticeps*.

Predictor variable	Function 1	Function 2
Head width	0.008	1.368
Head length	−0.286	−0.613
Maximum ocular diameter	0.076	0.412
Scape length	−0.058	0.304
Pronotal width	−0.031	0.276
Petiole width	−0.218	−0.289
Post-petiole width	−0.003	−0.066
Thorax length	−0.266	−0.554
Hind femur length	−0.022	0.532
Gastral tergite width	1.438	−0.664

See also Tables 1 and 2.

We performed a morphometric analysis of the female castes using multivariate analysis-of-variance (MANOVA). The data set included 10 continuous external characters; ocellus diameter was treated as a discrete variable because workers lack ocelli (see below). An *a posteriori* univariate F test was used to determine which variables contributed to overall differences among the castes [13]. We then assessed degree of overlap among the castes by performing a discriminant analysis using these 10 characters. The discriminant analysis developed predictive discriminant functions for each caste, which were then applied to all individuals during the same execution of the model [13]. The 10 characters were entered into the model simultaneously using caste as the grouping variable. The model used *a priori* classification, and prior probabilities were computed from group sizes. Two of the discrete characters (ovariole number and diameter of the anterior ocellus) were compared across castes using a Kruskal-Wallis test [14].

**Table 3:** Number of female sexuals of *Pogonomyrmex laticeps* collected during February 2010 at two localities (Punta Balasto RAJ #4363–4388; Chilecito RAJ #4404–4413; BQ = brachypterous queens, EQ = ergatoid queens, d = dealate).

Colony code	Queen pupae	Callow queens	Fullypigmentedqueens	Males	Workers	Mating status & ovary data
RAJ 4363	13 BQ	0	1 BQ (d)	13	>111	Mated, 12 ovarioles, many yellow bodies
RAJ 4366	0	6 BQ	0	1	>196	Virgin, 15 ovarioles (*n* = 6)
RAJ 4370	4	0	0	0	>87	ND
RAJ 4383	0	14 BQ	0	0	>179	ND
RAJ 4385	0	5 BQ	1 BQ (d)	0	>186	Not dissected
RAJ 4386	0	4 BQ	0	0	>44	ND
RAJ 4388	1 BQ	52 BQ	2 BQ (1d)	30	ND*	ND
RAJ 4404	0	0	>20 EQ	15	ND	Virgin, 12–14 ovarioles (*n* = 4)
RAJ 4412	0	0	10 EQ	9	ND	Virgin (*n* = 2)
RAJ 4413	0	0	>50 EQ	14	ND	Virgin, 14 ovarioles (*n* = 2)

Another six colonies from Punta Balasto yielded no sexuals. Colony codes refer to R.A. Johnson collection numbers.

* ND = No data.

We estimated relative wing size for two species with brachypterous queens (*P. laticeps* and *P. huachucanus*) and two species with normal winged queens (*P. inermis* and *P. uruguayensis*) that are close relatives of *P. laticeps* (preliminary phylogenetic data; C. Moreau and R.A. Johnson, unpublished). Relative wing size was calculated as wing length/mesosoma length, and the values were compared across species using a one-way ANOVA.

**Figure 6:**
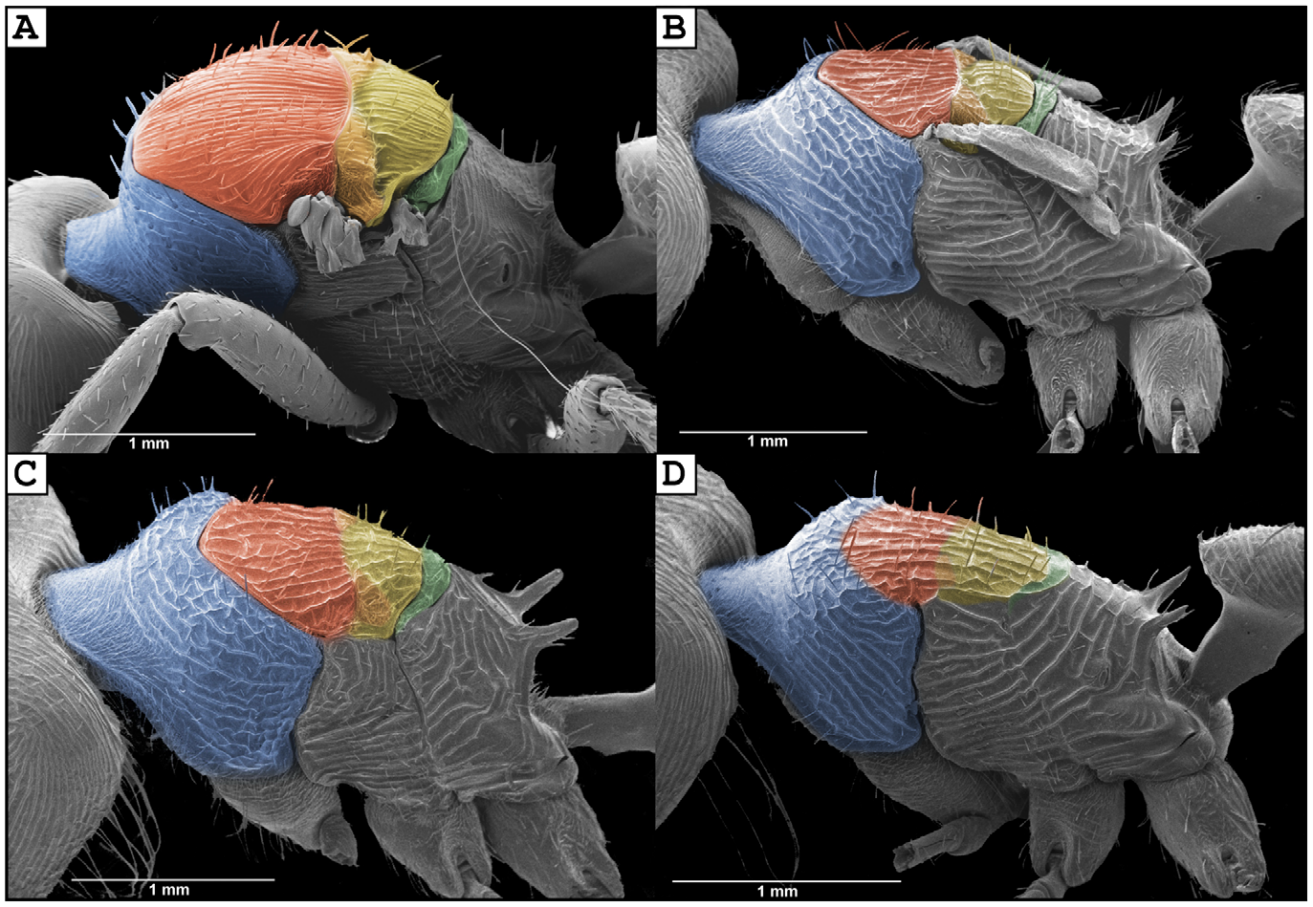
Scanning electron micrographs showing the different degrees of fusion of the dorsal thoracic sclerites for the three female castes of *Pogonomyrmex laticeps* in comparison with normal winged queens. (A) normal winged queen of *P. uruguayensis* (wings removed to show thorax), (B) brachypterous queen, (C) ergatoid queen, (D) worker. Blue = pronotum; red-orange and yellow = mesonotum; green = metanotum.

**Figure 7:**
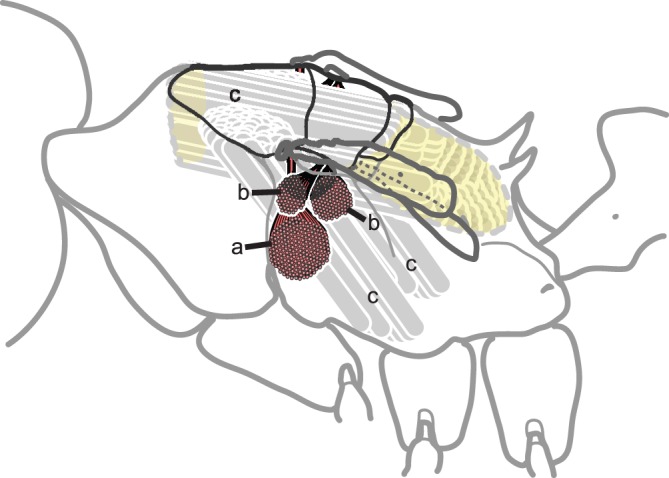
Brachypterous queens of *Pogonomyrmex laticeps* have a full set of direct flight muscles that tilt and fold the wings (red), but lack the phragmata and indirect muscles that power flight. (a) = wing deflector (basalare muscle), (b) = wing flexors (third axillary muscles), (c) = position of the phragmata (yellow) and indirect flight muscles (grey) in the thoracic cavity as they would occur in fully-winged queens.

We dissected the thorax of 3–4 individuals per caste to determine presence/absence of flight muscles and to further assess the degree of fusion/articulation between the thoracic sclerites. Specimens that had been stored in 70% ethanol were disarticulated with microforceps, stained for 1 h in a 0.2% aqueous solution of methylene blue, returned to 70% ethanol, then examined under a dissecting microscope. We considered two sclerites to be articulated (mobile) if we could cleanly isolate them from each other by gently pulling them apart, and sutured (immobile) if pulling resulted in an uneven break of their margins.

We also took scanning electron microscope (SEM) micrographs of the thorax ( = mesosoma) of workers, ergatoid queens, and brachypterous queens of *P. laticeps*, and normal winged queens of *P. uruguayensis,* in order to compare the degree of sclerite fusion. Whole specimens were fixed in 70% ethanol, air-dried, then mounted on SEM stubs using conducting double-sided adhesive tape. After sputter-coating with gold-palladium, images were taken with a Cambridge S260 microscope.

### Queen-worker Dimorphism and Fat Content

Dry mass was measured by collecting five ergatoid queens and five workers from each of three Chilecito colonies, as well as six brachypterous queens from Punta Balasto; brachypterous queens had been previously stored in 95% ethanol. Individuals were placed in an oven at 50–55° C for >72 h, then weighed. Dry mass of queens and workers was averaged within each colony, and these mean colony values were used to calculate the grand mean. Likewise, the queen to worker mass ratio was calculated for each colony, then averaged across colonies.

Total fat content was determined for six fully-pigmented ergatoid gynes. Individuals were dried at 55° C for >72 h, weighed, and then each individual was placed in a vial of petroleum ether (boiling point 30–60° C). Individuals were crushed to expose fat bodies and remained in ether for >24 h. The contents of each vial were rinsed through filter paper, dried, and weighed to 0.01 mg. Percent total fat content was calculated using the formula: 100×(DM – FFDM)/DM, where DM is dry mass and FFDM is fat free dry mass. All brachypterous gynes that we collected were callows (i.e. recently emerged), such that total fat content was not determined.

Voucher specimens are deposited in the R.A. Johnson collection, Tempe, AZ, USA [RAJC], and the Museo Argentino de Ciencias Naturales “Bernardino Rivadavia”, Buenos Aires, Argentina [MACN].

## Results

### Morphometric Comparison of the Female Castes

Sexual castes were conspicuous during excavations at both sites: queens at Punta Balasto were callows (i.e., pale coloration) with tiny wings (Figure 2), while ergatoid queens at Chilecito were noticeably larger than workers (Figures 3, 4). These observations were confirmed by our morphometric analysis, which demonstrated that size of the female castes differed significantly (MANOVA: Box’s M test, F_165, 10878 df_ = 1.19, *P* = 0.051; Wilks’ λ = 0.017, F_30, 241_ = 24.4, *P* << 0.0001); all 10 continuous characters varied among castes (univariate F-tests within MANOVA, *P* < 0.0001; Table 1). Eight of the 10 characters showed the same pattern of variation: workers from Chilecito were the smallest in size, workers from Punta Balasto were significantly larger, ergatoid queens were significantly larger than workers from both sites, and brachypterous queens were significantly larger than all others (Duncan’s multiple range test, *P* < 0.05). However, workers from Punta Balasto and ergatoid queens (Chilecito) were not different in size for scape length and hind femur length, and they were intermediate to both other groups) (Table 1).

Discriminant analysis correctly classified 94.7% (90 of 95) of all individuals. One worker from each site was misclassified as a worker from the other site, one ergatoid queen was misclassified as brachypterous, and two brachypterous queens were misclassified as ergatoid queens. The standardized coefficients of the canonical functions indicated that tergite width was the primary contributor to discriminant function 1, accounting for 90.5% of the variance. Head width was the primary contributor to discriminant function 2, accounting for 8.0% of the remaining variance (Table 2, Figure 5); accordingly head width is much less important than tergite width as a caste difference.

Both ergatoid and brachypterous queens had a spermatheca and 12–15 ovarioles, whereas workers lacked a spermatheca and had two ovarioles (Kruskal-Wallis test, *P* < 0.01; Tables 1 & 3). Both queen phenotypes had rudimentary ocelli that were similar in size, while workers lacked ocelli (Kruskal-Wallis test, *P* < 0.01; Table 1).

### Caste Variation in Thorax Morphology and Wing Musculature

The dorsal thoracic sclerites were completely fused in workers, while there was a clear boundary (suture) between the pronotum, mesonotum, and metanotum in ergatoid and brachypterous queens (Figures 2, 3, 4, 6). These sclerites were fused and immobile (i.e. sutured) in ergatoid queens whereas in brachypterous queens they were fully articulated. The mesonotum of the latter is shorter compared to the normal winged queens of *P. uruguayensis,* and it does not appear “bulged” in profile (Figure 6A,B). As a consequence, the slope at which the pronotum raises to meet the mesonotum is less steep: it is fully vertical in normal winged queens, while it rises at an approximately 45° angle in brachypterous queens (as it does in ergatoid queens and workers) (Figure 6). Ergatoid queens also exhibited a continuum in the simplification and fusion of the thorax. The mesonotum was undivided in most specimens (Figure 6C), whereas in others a distinct transscutal fissure divided the mesonotum in two parts (scutellum and scutum). Both ergatoid queen forms occurred within the same colony. The region where the forewings normally attach was simplified and immobile in ergatoid queens (sutured), while the wing articulation was mobile and normally developed in brachypterous queens.

Both queen phenotypes lacked indirect wing muscles and any trace of the internal phragmata; the latter are cuticular projections present in all flying insects and function to support the longitudinal wing muscles. Unlike ergatoid queens, brachypterous queens had a fully developed set of direct flight muscles, which were attached to the base of the tiny forewings (Figure 7); these muscles serve as either deflectors or flexors (“tilting” movements) for the wings but do not power flight. The action of these muscles was confirmed when the forewings moved after gently pulling the tendons on partially dissected individuals. The significance (if any) of these muscles in *P. laticeps* is unknown.

### Production of Sexuals

Gynes and males were found in most colonies at this time of year. Punta Balasto colonies produced 1–52 brachypterous queens (either as pupae or callows); all callow queens had undeveloped ovarioles (Table 3). We also collected several fully pigmented ‘dealate’ queens (i.e. wing bases clearly seen) that were inseminated and with active ovaries. In Chilecito, the upper (watered) regions of the three excavated colonies contained 10 to more than 50 ergatoid queens.

### Queen to Worker Dimorphism and Fat Content

Dry mass averaged 7.57 ± 0.10 mg and 2.99 ± 0.20 mg for ergatoid queens and workers, respectively, yielding a queen to worker dry mass ratio of 2.55 ± 0.14. These ergatoid queens had 25.78 ± 2.22% total fat content (*n* = 6 queens from 3 colonies), which is consistent with finding large fat bodies in their gasters. The dry mass of brachypterous queens (*n* = 6) averaged 3.51 ± 0.06 mg, but this is an underestimate due to prior storage in 95% ethanol. Moreover, all brachypterous gynes were callow and thus younger than the ergatoid gynes, thus they had had less time to accumulate fat stores in their natal nests. We assume that non-callow brachypterous queens are heavier than ergatoid queens given that the former were larger for nine of our 10 external morphological characters (all but ocellus diameter).

## Discussion

### Queen Morphology

Many ants have evolved non-flying queens as a replacement for the ancestral winged queens [5], but *P. laticeps* is highly unusual because both brachypterous and ergatoid queens exist. Although these two queen phenotypes have a reduced thorax that is more or less worker-like, their gasters have considerably larger volume than that of workers (Table 1, Figure 5). This emphasizes that non-flying queens remain specialized for egg-laying. The brachypterous queens of *P. laticeps* were significantly larger than the ergatoid queens in all external measures except for size of the anterior ocellus. However both queen phenotypes have comparable egg-laying potentials, i.e., a similar number of ovarioles, which is not always the case for ants with dimorphic queens [5], [9].

Although they cannot fly, brachypterous queens in *P. laticeps* share several morphological characters with normal winged queens: their flight sclerites are articulated and separable, and they retain direct flight muscles. The internal phragmata are cuticular structures that are fixed during adult life, and their absence in brachypterous queens is unmistakable evidence that indirect wing muscles never develop (i.e. the absence of wing muscles cannot be due to post-emergence histolysis). Their wings are minute however (Figure 2), much shorter than in brachypterous winged congeners – forewings are two-thirds of normal length in *P. huachucanus* [15] and one-half normal length in *P. andinus* [10]. The wing length to mesosoma length ratio was lowest in *P. laticeps* (0.42), intermediate in *P. huachucanus* (1.69), and highest in the two related normal winged species, *P. inermis* (2.25) and *P. uruguayensis* (2.38); this difference is significant (F_3,29_ = 599.7, P < 0.001, Duncan’s range test, P < 0.001). In *P. huachucanus,* laboratory observations indicated that brachypterous queens shed their wings by the time they acquire adult pigmentation [15], and the same appears true in *P. laticeps*.

The absence of the indirect flight muscles in brachypterous queens is also reflected in the shape and size of the dorsal thoracic plates. The mesonotum is flattened in profile while the thoracic box is strikingly reduced in volume when compared with normal winged queens of *P. uruguayensis* (Figure 6A,B). Ergatoid queens have a more simplified thorax, but there was variation in the extent of sclerite fusion. This is also seen in the ergatoid queens of *P. pima* and *P. imberbiculus* [6], [7]. The absence of indirect flight muscles in both phenotypes results in a significant reduction in the production cost of gynes, because these muscles make up a minimum of 12–16% to as much as 55% of the total dry mass [16]; moreover the high metabolic cost of maintaining these muscles is eliminated. We also documented a low queen to worker mass ratio (see below), which is further evidence for a low *per capita* cost of gynes.

Ocelli are involved in stabilization reflexes during flight [17]. Non-flying ant queens often have ocelli with various degrees of reduction in size, number, or pigmentation, and they lack a corneal lens. Rudimentary ocelli suggest partial activation of the winged queen developmental pathway. That the ocelli in *P. laticeps* are rudimentary is evidenced by their small size (mean diameter = 0.05 mm for both phenotypes [see also 10], which is 0.02% of head width) compared to the larger ocelli (mean diameter = 0.06 mm, 0.05% of head width) in the small flying queens of *P. pima* [7].

Although workers from our two sites differed in size (Table 1, Figure 5), detailed examination of their morphology, as well as that of males, suggests that we studied only one species. Colonies at each site only produced one queen phenotype; mated brachypterous queens were found at Punta Balasto, indicating that they produced homotype gynes. Future studies should examine the phylogeography of *P. laticeps* and map the fragmented geographic distribution (Figure 1) of the two queen phenotypes.

In sharp contrast to *P. laticeps*, both brachypterous and ergatoid queens occur in the same colonies of *Cataglyphis floricola* [12]. Brachypterous queens are considerably larger and heavier than ergatoid queens (fresh masses are 6.5 ± 0.2 and 3.7 ± 0.3 mg, respectively), and they have twice as many ovarioles. Colonies of *C*. *floricola* are monogynous and produce more ergatoid gynes (2.4 ± 0.5) than brachypterous gynes (0.74 ± 0.2) [12]. In conclusion, *C*. *floricola* ergatoids are much cheaper but also less fertile than brachypterous queens, which is intriguing given that both phenotypes disperse with nestmate workers to found a new colony, i.e. dependent colony founding (DCF) [12]. Given that such queens rely entirely on workers, this removes constraints on their morphology, which is not the case in *P. laticeps* (see below).

### Colony Founding

Typically, ant species with ergatoid queens reproduce using DCF, i.e., queens cannot start a new colony alone [5], [18]. However ergatoid foundresses are solitary in a few known exceptions: one species in each of the subfamilies Ponerinae [19], [20] and Myrmeciinae [24], as well as three species of *Pogonomyrmex* [15]. Investment in gynes is one trait that differs between DCF and independent colony founding (ICF) species. The latter produce large numbers of gynes annually, while DCF species produce very few gynes [12], [18], [21]. We do not know what colony founding strategy is used in *P. laticeps*, but our observation that dozens of gynes were produced in colonies of either queen phenotype (Table 3) suggests that both phenotypes use ICF. Correspondingly, numerous gynes are produced in one brachypterous congener (*P. huachucanus*) and two ergatoid queen congeners (*P. cunicularius cunicularius* and *P. cunicularius pencosensis*) in which ICF has been observed [15]; *P*. *huachucanus* colonies produce >50 gynes (R.A. Johnson, unpublished data) while colonies of P. *cunicularius pencosensis* and *P. cunicularius cunicularius* produce >100 gynes (R.A. Johnson and C. Peeters, unpublished data).

If *P. laticeps* initiate nests using ICF, then foundresses (ergatoid or brachypterous) are undoubtedly obligate foragers. Indirect evidence for this prediction is the queen to worker mass ratio and fat content, which are both among the lowest known for species of *Pogonomyrmex*. Fat content was lower than all free-living North American congeners [22; R.A. Johnson, unpub. data]. Similarly, the queen to worker mass ratio (2.55) for ergatoid queens of *P. laticeps* was similar to that of *P. pima* (2.05) and *P. montanus* (2.78); both of these species are non-claustral (R.A. Johnson, unpublished data). The majority of myrmicine ants are fully claustral founders (ICF), but there have been sporadic reversions to non-claustral ICF, including in several *Pogonomyrmex* species with winged queens [22]. The success of foraging queens in *Pogonomyrmex* is enhanced by the synchrony between colony founding and the seasonal production of abundant seeds; such predictable resources exceed any possible metabolic reserves. Hence, obligate foraging during ICF brings benefits that offset the tradeoff between solitary dispersal and low survival. Importantly, more and/or heavier minims (nanitic workers) can be produced relative to claustral species with fixed energetic reserves [23].

The existence of different non-flying phenotypes in two populations of *P. laticeps* suggests that the ancestral winged queens were replaced independently. Similarly, either ergatoid or brachypterous queens exist in congeneric species of *Myrmecia* [*M. pulchra* and *M. regularis*; 24,25] and *Aphaenogaster* [*A. araneoides* and *A. senilis*; 26,27] which is further evidence of alternative solutions to the selective pressures against flying queens in ants. W.M. Wheeler [28] hinted that brachypterous and ergatoid queens are sequential stages during the evolutionary loss of wings, but we find no support for this. Importantly, these novel queen phenotypes always uncouple growth of the thorax and gaster, and thereby decrease the *per capita* cost of producing gynes.

## References

[1] Wagner DL, Liebherr JK (1992) Flightlessness in insects. Trends in Ecology and Evolution 7: 216–220. 21236012 10.1016/0169-5347(92)90047-F

[2] Zera AJ, Denno RF (1997) Physiology and ecology of dispersal polymorphism in insects. Annual Review of Entomology 42: 207–230. 10.1146/annurev.ento.42.1.207 15012313

[3] Guerra PA (2011) Evaluating the life-history trade-off between dispersal capability and reproduction in wing dimorphic insects: a meta-analysis. Biological Reviews 86: 813–835. 21199288 10.1111/j.1469-185X.2010.00172.x

[4] Zera AJ, Harshman LG (2001) The physiology of life history trade-offs in animals. Annual Review of Ecology and Systematics 32: 95–126.

[5] Peeters C (2012) Convergent evolution of wingless reproductives across all subfamilies of ants, and sporadic loss of winged queens (Hymenoptera: Formicidae). Myrmecological News 16: 75–91.

[6] Heinze J, Hölldobler B, Cover SP (1992) Queen polymorphism in the North American harvester ant, *Ephebomyrmex imberbiculus* . Insectes Sociaux 39: 267–273.

[7] Johnson RA, Holbrook CT, Strehl C, Gadau J (2007) Population and colony structure and morphometrics in the queen dimorphic harvester ant, *Pogonomyrmex pima* . Insectes Sociaux 54: 77–86.

[8] Buschinger A, Heinze J (1992) Polymorphism of female reproductives in ants. In: Billen J, editor. Biology and Evolution of Social Insects. Leuven, Belgium: Leuven University Press. 11–23.

[9] Heinze J, Tsuji K (1995) Ant reproductive strategies. Researches on Population Ecology 37: 135–149.

[10] Kusnezov N (1951) El género *Pogonomyrmex* Mayr (Hym., Formicidae). Acta Zoologica Lilloana 11: 227–333.

[11] Cuezzo F, Claver S (2009) Two new species of the ant genus *Pogonomyrmex* (Hymenoptera: Formicidae) from Argentina. Revista de la Sociedad Entomológica Argentina 68: 97–106.

[12] Amor F, Ortega P, Jowers MJ, Cerda X, Billen J, et al (2011) The evolution of worker-queen polymorphism in *Cataglyphis* ants: interplay between individual- and colony-level selections. Behavioral Ecology and Sociobiology 65: 1473–1482.

[13] SPSS I (1990) SPSS Reference Guide. Chicago: SPSS, Inc. 949 p.

[14] Siegel S, Castellan NJ (1988) Nonparametric Statistics for the Behavioral Sciences. New York: McGraw-Hill. 399 p.

[15] Johnson RA (2010) Independent colony founding by ergatoid queens in the ant genus *Pogonomyrmex*: queen foraging provides an alternative to dependent colony founding. Insectes Sociaux 57: 169–176.

[16] Marden JH (2000) Variability in the size, composition, and function of insect flight muscles. Annual Review of Physiology 62: 157–178. 10.1146/annurev.physiol.62.1.157 10845088

[17] Krapp HG (2009) Ocelli. Current Biology 19: 435–437. 10.1016/j.cub.2009.03.034 19515345

[18] Peeters C, Molet M (2010) Colonial reproduction and life histories. In: Lach L, Parr C, Abbott K, editors. Ant Ecology. Oxford: Oxford University Press. 161–178.

[19] Villet M (1999) Reproductive behaviour of *Plectroctena mandibularis* F. Smith (Hymenoptera: Formicidae), a ponerine ant with ergatoid queens. African Entomology 7: 289–291.

[20] Villet M (1991) Colony foundation in *Plectroctena mandibularis* F. Smith, and the evolution of ergatoid queens in *Plectroctena* (Hymenoptera: Formicidae). Journal of Natural History 25: 979–983.

[21] Kronauer DJC (2009) Recent advances in army ant biology. Myrmecological News 12: 51–65.

[22] Johnson RA (2002) Semi-claustral colony founding in the seed-harvester ant *Pogonomyrmex californicus*: a comparative analysis of founding strategies. Oecologia 132: 60–67. 28547285 10.1007/s00442-002-0960-2

[23] Johnson RA (2006) Capital and income breeding and the evolution of colony founding strategies in ants. Insectes Sociaux 53: 316–322.

[24] Haskins CP, Haskins EF (1955) The pattern of colony foundation in the archaic ant *Myrmecia regularis* . Insectes Sociaux 2: 115–126.

[25] Clark J (1951) The Formicidae of Australia, vol. 1: Subfamily Myrmeciinae. Melbourne, Australia: Commonwealth Scientific and Industrial Research Organization. 203 p.

[26] Tinaut A, Ruano F (1992) Braquipterismo y apterismo en formicidos. Morfologia y biometria en las hembras de especies ibericas de vida libre (Hymenoptera: Formicidae). Graellsia 48: 121–131.

[27] Longino JT, Cover S (2004) A revision of the *Aphaenogaster phalangium* complex (Hymenoptera: Formicidae: Myrmicinae). Zootaxa 655: 1–12.

[28] Wheeler WM (1917) The phylogenetic development of subapterous and apterous castes in the Formicidae. Proceedings of the National Academy of Sciences 3: 109–117. 10.1073/pnas.3.2.109 PMC1091187 16586692

